# Investigating the feasibility and safety of transcranial infraslow gray noise stimulation as a potential treatment for generalized anxiety disorder

**DOI:** 10.1038/s41598-025-27624-3

**Published:** 2025-12-17

**Authors:** Cindy van Sleeuwen, Divya Adhia, Paul Glue, Dirk De Ridder

**Affiliations:** 1 Department of Surgical Sciences, Dunedin School of Medicine, University of Otago, Dunedin, New Zealand; 2 Department of Psychological Medicine, Dunedin School of Medicine, University of Otago, Dunedin, New Zealand; 3 Section of Neurosurgery, Department of Surgical Sciences, Dunedin School of Medicine, University of Otago, PO Box 56, Dunedin, 9054 New Zealand

**Keywords:** Anxiety, Electroencephalography, Stimulation, Neuromodulation, Insula, Emotion, Neuroscience, Health care, Public health, Quality of life, Therapeutics

## Abstract

Anxiety is a beneficial behavior that assists survival, although when high levels of anxiety persist it can hinder normal functioning. Despite numerous treatment options most individuals with Generalized Anxiety Disorder (GAD) continue to suffer from the disorder. A novel neuromodulatory treatment that safely and non-invasively alters pathological neural circuitry associated with GAD is required. Therefore, this study investigates the feasibility, safety, and effect of a new innovative High-Definition Transcranial Infraslow Gray Noise Stimulation (HD-tIGNS) targeting the anxiety network in people with GAD, in a delayed-start double-blinded randomized sham-controlled pilot trial (*N* = 22). HD-tIGNS was applied three times per week for three [delayed-start (following 3-weeks of actisham)], or six weeks (early-start). Anxiety questionnaires and electroencephalography were taken at baseline, mid-treatment, and post-treatment. On average, six participants were recruited per month with a mean treatment adherence of 99.5%, and an 8.3% dropout rate of enrolled participants. Clinically meaningful changes in GAD-7 were observed in 45% (early-start group) and 36% (delayed-start group) of participants following 6-weeks and 3-weeks of intervention respectively. No significant differences in brain activity or functional connectivity were observed. This study provides evidence supporting HD-tIGNS as a safe and feasible treatment approach for GAD, however future research should consider alternate network targets.

**Supplementary Information:**

The online version contains supplementary material available at 10.1038/s41598-025-27624-3.

## Introduction

Anxiety is a beneficial emotion that assists survival by appropriately enhancing our performance in predicted challenging situations, although when high levels of anxiety persist it can hinder normal functioning^1^. Generalized Anxiety Disorder (GAD) is one of the most common anxiety disorders and is associated with psychological, social, emotional, and physical dysfunction, with a lifetime prevalence of 5–7% and an annual prevalence of 2.9% (two-thirds female)^2–8^. GAD’s impact on everyday life is best explained by cognitive control impairments whereby negative stimuli persevere in one’s thoughts, in turn depleting neurocognitive resources^9,10^. For example, Jenkins et al.^9^ presented evidence supporting heightened response monitoring (demonstrated by heightened error-related negativity) in the medial prefrontal cortex in participants with major depressive disorder and comorbid anxiety, and proposed this was increased salience network (SN) activity compensating for lower central executive network (CEN) activity^9^. Moreover, Yoon et al.^10^ observed a diminished working memory capacity in GAD patients such that participants were less able to attend to task-relevant information when subjected to negative distractors during a reading span task^10^. These examples suggest inefficiency in utilizing neurocognitive resources in anxious individuals.

Existing treatments for GAD aim to reduce symptoms and restore function. However, most individuals with GAD continue to suffer from the disorder due to poor treatment compliance and long waitlists for psychological treatments^8,11–13^. Pharmacological treatments such as SSRI’s and SNRI’s are most prescribed as these medications have the highest response rates, typically reported around 50–75%, and are less resource intensive than psychological treatments^8,13^. Discontinuation rates for SSRI’s and SNRI’s have been reported as high as 46%, likely due to side effects such as weight gain, sexual dysfunction, and sleep disturbances^8,13^. Moreover, pharmacological treatments need to be gradually introduced to avoid adverse effects and initial increases in anxiety as these issues may compromise adherence^8,11^. If SSRI’s are not tolerated or are ineffective, agomelatine and pregabalin are recommended^8,11^. However, agomelatine is not registered for use in Australia nor is it available in New Zealand, thus availability issues for certain medications are known to exist^8,11^. Psychological treatments such as cognative behavioural therapy (CBT) are other first-line treatments for anxiety, demonstrating high response rates up to 66%, although discontinuation rates fall between 9 and 21%^13^. Reasons for discontinuing CBT include low motivation, poor readiness for change, and preference for alternate treatments^8,12,13^. In general, the number needed to treat GAD with antidepressants is 5.15^14^ and 6.3 for psychotherapy^15^, meaning that if five to six patients are treated, one improves better than placebo. Currently, there is no universally effective treatment for GAD^13^, and recent disinterest in research investments from pharmaceutical companies into brain-related disorders mean there is growing interest in other treatment modalities such as neuromodulation. Recent advances in neuroimaging have allowed for increased understanding of neuropathological markers underlying not just anxiety, but a multitude of clinical psychopathologies. This understanding might have potential for future curative interventions such that neuromodulatory treatment techniques have been suggested as possible gold standard treatments of the future^16–18^. On this notion, a novel neuromodulatory treatment that safely and non-invasively alters the pathological neural circuitry associated with GAD is required^13,17^.

Oscillations in the infraslow frequency (0.1 Hz) have gained increasing interest in recent years since they have been observed in fMRI blood oxygen level dependent (BOLD) signals in both cortical and subcortical networks^19–21^. Evidence suggests that these fMRI BOLD infraslow signals directly correlate with infraslow electroencephalography (EEG) oscillations^19–21^. Moreover, higher frequency bands such as beta and gamma are observed to be nested with infraslow fluctuations^20,22^. Infraslow oscillations might therefore be of interest as potential discriminatory markers between healthy and clinically diagnosed individuals. Studies investigating functional connectivity (FC) changes in GAD have implicated the involvement of multiple brain regions, specifically the amygdalae^5,12,23–25^, right dorsal anterior cingulate cortex (rdACC)^25–28^, insulae^23,29,30^, bilateral orbito-frontal cortex (OFC)^25,31^, parahippocampi^25,32^, and bilateral subgenual anterior cingulate cortex (sgACC)^9,33,34^. These regions are linked to emotional and autonomic dysregulation, and somatic health problems observed in GAD^3,5,6,35^. Recent investigations into graph theory underlying anxiety show overall lower global efficiency and altered local network functioning^36,37^. Thus, the main brain regions targeted in this study include amygdalae, rdACC, insulae, bilateral OFC, parahippocampi, and sgACC.

Mental health disorders (including GAD) impose a large and increasing (16.7% between 1990 and 2021)^38^ societal burden with many countries facing mental health crises and calling for non-pharmacological treatment approaches, whereby novel non-invasive neuromodulatory interventions are thought to be the treatment of future^16–18^. This is a growing notion in the field of healthcare, with recent literature showing that transcranial electrical stimulation (TES) modalities have been observed to effectively modulate FC in the brain as compared to sham stimulation^39,40^. Specifically, prefrontal transcranial direct current stimulation (tDCS) was observed to improve cognitive performance in healthy populations and individuals with neurological conditions^39^. Moreover, transcranial random noise stimulation (tRNS) has been hypothesized to be more effective than other non-invasive neuromodulation techniques such as tDCS^41^. Noise is an essential natural phenomenon present in all scale free systems, for example, synaptic noise and stochastic activity of neuronal signals/ion channels in the human brain^42–46^. TRNS (i.e., a Gaussian distribution of white noise) has been most extensively studied^47,48^ in learning^49–52^, and in clinical entities such as pain^53,54^ and tinnitus^55–57^. TRNS is superior to tACS and tDCS in tinnitus suppression^57^ and has been suggested to be a promising novel approach for treatment and prevention of various diseases^40,49,58–61^. Repeated tRNS can induce lasting modulatory effects on neuronal activity via changes in mechanisms such as repeated opening of sodium channels (under the stochastic resonance phenomenon) and calcium-dependent synaptic plasticity in glutamatergic neurons^40,62–64^.

Structured noise may be superior to induce stochastic resonance than unstructured white noise to modulate the human brain^65,66^. White noise is purely chaotic and contains no information as all frequencies are characterized by equal power, whereas structured noise is defined by colors, and described by its power to frequency analysis in which the power exponent β describes the steepness of the slope. 1/f power means that low frequencies have high power and high frequencies have low power. This is typical of EEG recordings^44–46^. In other words, there is more power in the higher frequencies than lower frequencies, which theoretically may help disrupt increased FC^18,42^. Transcranial structured noise stimulation is hypothesized to strengthen physiological 1/f activity via stochastic resonance effect, yet at higher amplitudes might disrupt FC. Previous investigation of transcranial structured noise stimulation has been found to safely modulate brain activity and connectivity in food addiction, chronic low back pain, and tinnitus^40,58,67^. Gray noise is a combination of 1/f^β^ and 1/f^-β^, which permits the generation of a trough at 10 Hz (Fig. 2). The gray noise spectrum thus has higher power in both the lower frequency and higher frequency bands and could be superior to the other tRNS techniques. Further, to resemble the physiological nested oscillation of the brain waves, where higher frequencies are overlaid on lower frequencies, we overlaid the gray noise on a sine infraslow (0.1 Hz) wave to create the infraslow gray noise stimulation (IGNS) design. With the help of Neuroelectrics (Barcelona, Spain) and their Stimweaver simulation software (STIMWEAVER SPRxxx, www.neuroelectrics.com/products/research/modeling-services), we created a ‘high definition’ montage that could precisely target the brain regions of interest. Thus, the High-Definition Transcranial Infraslow Gray Noise Stimulation (HD-tIGNS) design aims to promote emotional regulation and normalize brain-wide FC by strengthening normal physiological activity in the aforementioned brain regions in the low frequencies (up to 10 Hz), and disrupt the beta and gamma activity which are commonly present in GAD^20,22^.

We hypothesized that specifically and simultaneously targeting the key nodes of the anxiety networks using a novel HD-tIGNS technique might induce neuroplastic changes by normalizing brain-wide FC, promoting greater emotional regulation and producing meaningful clinical benefits. Therefore, the aim of this study was to evaluate the proof-of-concept that a novel HD-tIGNS technique can safely alter brain activity (i.e., current source density) and the FC within the anxiety network and improve clinical anxiety outcomes. This study is the first to utilize HD-tIGNS for GAD in humans. The objectives of this study were to firstly evaluate the feasibility and safety of HD-tIGNS in individuals with GAD, and secondly, explore the effect of HD-tIGNS on clinical anxiety outcomes and brain activity and FC within and between key hubs of the anxiety network (insulae, amygdalae, subgenual anterior cingulate cortex extending into the orbitofrontal cortex (sgACC-OFC).

## Materials and methods

### Trial design and participants

This study is a delayed-start double-blinded (participant, outcome-assessor) randomized sham-controlled pilot trial conducted at the Dunedin School of Medicine laboratory, Otago University (New Zealand). A modest convenience sample was recruited from the community via local advertisements in Dunedin (*N* = 24, Female = 95.8%). Sample size calculation was not performed as this is a pilot trial. Participants were adults (18–60 years) diagnosed with GAD (participants were asked if they had ever been diagnosed with GAD by a health care professional, and if yes, when the diagnosis was made and by whom) and scored ≥ 24 on the Mini Mental State Examination (range = 0–30). For inclusion and exclusion criteria see Supplemental Fig. S1.

**Fig. 1:**
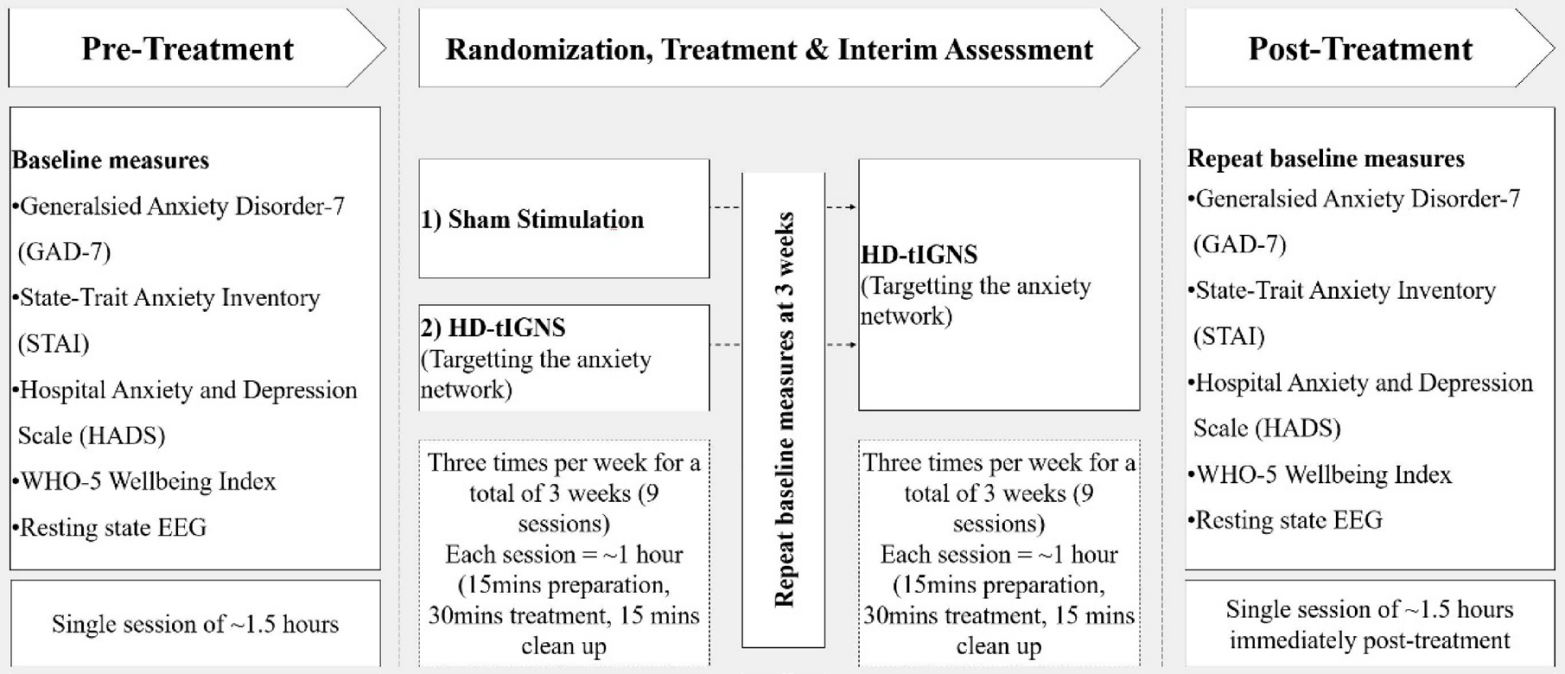
Study design and time-duration for each phase. EEG: electroencephalography; HD-tIGNS: high-definition transcranial infraslow gray noise stimulation.

Primary outcome measures consisted of feasibility and safety assessments [recruitment, drop out, and intervention adherence rates, and the Discontinuation-Emergent Sign and Symptom (DESS) questionnaire, respectively], collected throughout the study. Secondary exploratory outcomes consisted of three anxiety questionnaires [Generalized Anxiety Disorder Scale-7 (GAD-7), Hospital Anxiety and Depression Scale-Anxiety (HADSA), State-Trait Anxiety Inventory (STAI)], and resting-state EEG, collected at three time points: baseline intake session (T0), mid-treatment (T1), and post-treatment (T2). A staff member not involved in the study enrolled and randomized participants equally into two groups (1:1) based on age (blocks:18–30, 31–40, 41–50, 51–60 years), and GAD-7 score (blocks: 0–9, 10–14, ≥ 15), and notified the treatment administrator directly. The two groups were an early-start (ES) group (*n* = 11) who received HD-tIGNS for six consecutive weeks, and a delayed-start treatment control (DS) group (*n* = 11) who first received three weeks of actisham stimulation (i.e., active control condition) followed by HD-tIGNS for three weeks (Fig. 1). The delayed start study design was beneficial as it offered firstly, an ethical advantage as all participants were guaranteed the intervention and were thus more likely to remain committed throughout the study duration, and secondly, it allowed for the investigation of differences in the length of intervention (i.e., three vs six weeks of intervention). Participants and outcome assessors were blinded to group allocation. Participants were advised that the actisham and HD-tIGNS conditions would feel identical (i.e., they would feel stimulation at first and then become accustomed to sensation after a few minutes). At the mid-treatment (T1) and post-treatment (T2) measurement sessions, participants were asked to identify the type of treatment they believed they received and rate their confidence in this belief on a scale from 0 to 100% (0% = not at all confident, 100% = extremely confident). Participants were also asked to provide reasons for their judgement and confirm whether the intervention was revealed to them.

This study was approved by the Health and Disability Ethics Committees New Zealand (Ethics Reference: 2023FULL13910), and the trial was registered at the Australia New Zealand Clinical Trials Registry (registration number:ACTRN12623000250639; on 08/03/2023). All experiments were performed in accordance with the declaration of Helsinki. Oral and written informed consent was obtained from all participants prior to participation.

### Intervention

**Fig. 2:**
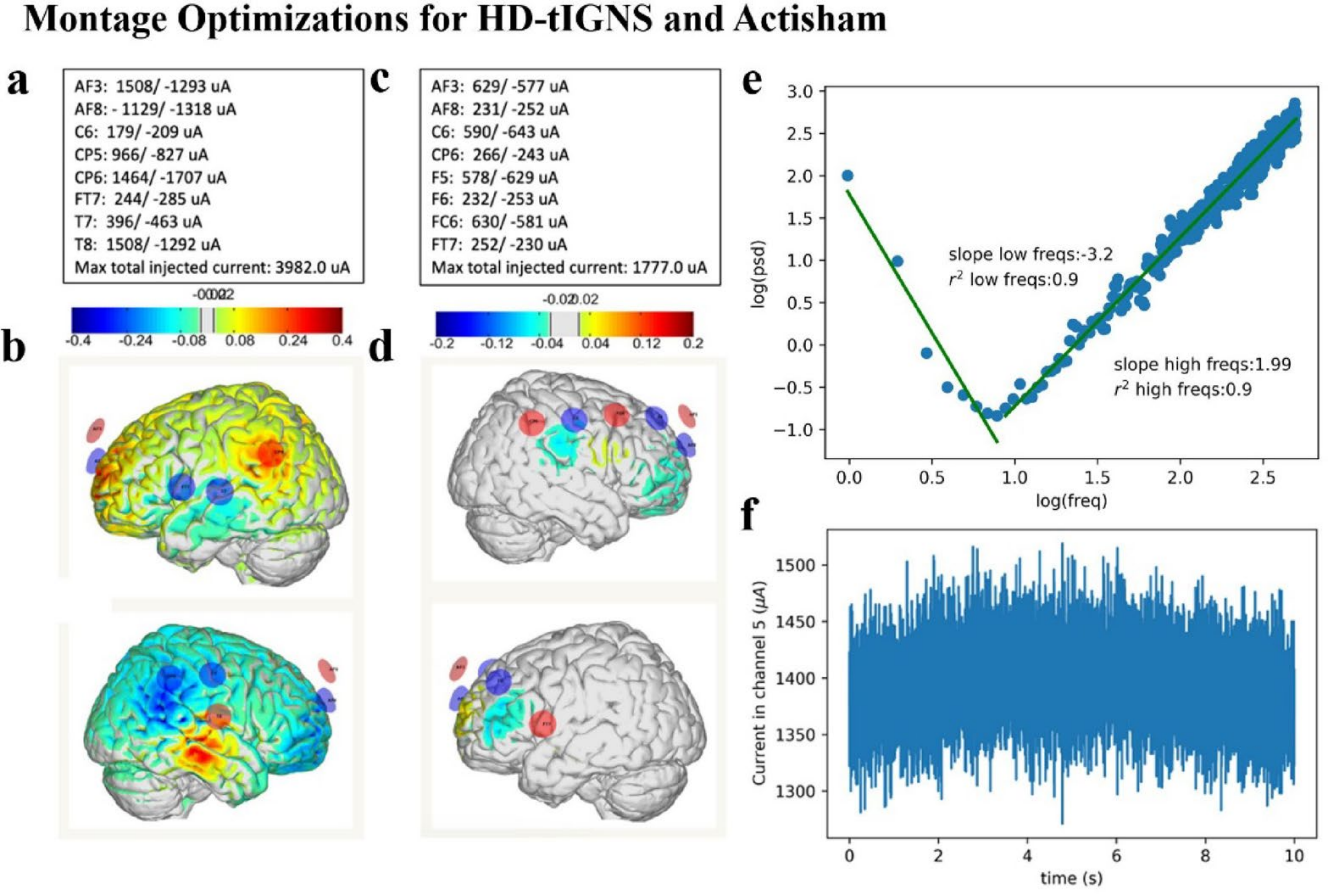
This figure shows the optimizations created by Neuroelectrics for the Starstim20 device. Pictured is the high-definition transcranial gray noise stimulation (HD-tIGNS) montage which targets the activity of the amygdalae, rdACC, insulae, and bilateral OFC, parahippocampus, and sgACC (**a**, **b**), and the actisham montage which mimics the sensation of the HD-tIGNS (**c**, **d**). The maximum and minimum electrical currents at each electrode and the maximum total injected current for HD-tIGNS (**a**) and actisham (**c**) are given. The distribution of the electric field [V/m] perpendicular to the cortical surface are shown, where blue corresponds to inhibition, red to excitation and gray to non-stimulation for HD-tIGNS (**b**) and actisham (**d**). The electrical current is delivered through the eight channels in a fractionated way, to optimize the current at the brain targets, based on a computer simulation on a standard head model. Also shown are the characteristics of the custom infraslow gray noise waveform (**e**, **f**). HD-tIGNS: high-definition transcranial infraslow gray noise stimulation.

Participants were comfortably reclined for the duration of each session where HD-tIGNS was administered for 30 min by a trained researcher using the Starstim20 (Neuroelectrics), a battery-driven, high-definition transcranial electrical stimulator for multitarget stimulation with 20 independent current sources. 20 electrodes (Neuroelectrics, code:NE029) were placed on a standardized 10-10EEG neoprene head cap. The stimulation waveform was delivered through eight electrodes at a current strength of maximum 1.5 mA per electrode (Fig. 2; 30 min including 60 s ramp-up and 60 s ramp-down), which adheres to safety guidelines^63,68,69^. The DS group received an actisham protocol (Neuroelectrics), where a reduced amount of current was applied for the first two minutes only (including 30 s ramp-up and 30 s ramp-down), creating a skin sensation designed to mimic the active stimulation allowing for adequate blinding (Fig. 2)^70^. This is sufficient to blind the participant as it mimics the HD-tIGNS condition where the scalp becomes accustomed to the sensation and desensitizes after a few minutes. While the actisham montage still applies current to the scalp, the phases and currents in the electrodes have been adjusted to maximize shunting through the scalp, resulting in average E-fields 5–18 times lower in all target brain regions (Fig. 2d)^70^.

### Outcome measures

#### Primary outcome measures

*Feasibility measures:* included rate of recruitment (average per month), percentage of participants recruited of the total number screened and their reasons for exclusion, number of dropouts, and treatment adherence (percentage of treatment sessions attended). *Safety:* The DESS Questionnaire was used to identify any adverse effects associated with HD-tIGNS at T1 and T2^71^. At T2 participants rated HD-tIGNS for acceptability, satisfaction, and effectiveness on an electronic self-reported numeric rating scale (0 = Not at all acceptable/satisfied/effective, 10 = Extremely acceptable/satisfied/effective), for example ‘Overall, how acceptable was the brain stimulation treatment to you?’.

#### Secondary (exploratory) clinical outcome measures

**Fig. 3:**
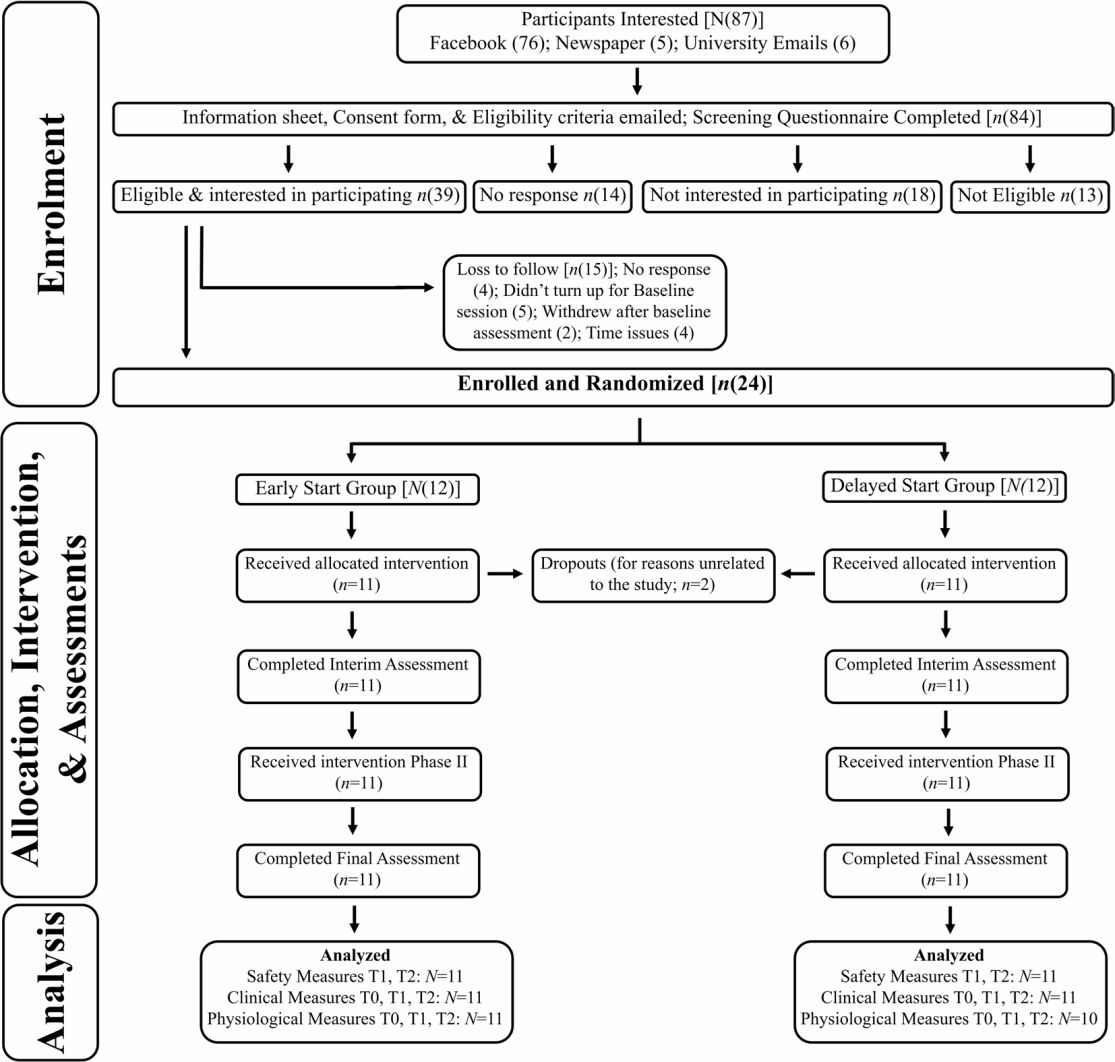
Participant flow diagram showing participant recruitment across study phases.

*Clinical Measures:* The GAD-7 is a validated, self-rated scale often used for screening GAD^72^. GAD-7 scores were calculated according to Spitzer et al. (2006; Range 0–21)^72^. The HADSA is a reliable, valid questionnaire subscale commonly used for screening anxiety^73^. The HADSA was scored according to Snaith (2003; range 0–21)^73^. The STAI is a commonly used measure of anxiety with two components (i.e., state [STAI-S], and trait [STAI-T] anxiety), and can be used to distinguish anxiety from depression (range 20–80 per component)^74^. *Physiological measures:* Resting-state EEG (10 min, eyes closed) was obtained as described by previous investigators^40^ using the SynAmps RT Amplifier EEG-recording system (Compumedics Neuroscan) with Compumedics Neuroscan Curry software (Version 8 XS, www.compumedics.com.au/en/products/curry/) at a sampling rate of 500 Hz. The 64 EEG electrodes were placed in standard 10–10 international placement, and impedances were checked to remain < 5 kΩ. Data was resampled to 128 Hz, band-pass filtered (fast Fourier transform filter) to 0.01 to 44 Hz, unwanted channels were removed (56 channels remaining after 8 channels were removed, namely ‘F11’, ‘F12’, ‘FT11’, ‘FT12’, ‘M1’, ‘M2’, ‘CB1’, ‘CB2’), and bad epochs were rejected using the EEGLAB package^75^ in MATLAB (MathWorks, Natick, MA). Thereafter, ICoN (Marco Congedo, 2018; sites.google.com/site/marco-congedo/science/software/icon) was used to perform independent component analysis (ICA). ICoN allows the ICA of EEG recordings to be visually inspected for artifacts, which were manually rejected. Flat channels were then interpolated using the EEGLAB package in MATLAB. This carefully inspected and cleaned data was organized into infraslow (0.08–0.12 Hz), delta (2–3.5 Hz), theta (4–7.5 Hz), alpha (8–12 Hz), beta (12.5–30 Hz), and gamma (30.5–44 Hz) frequency bands. Of note, no a priori hypothesis for physiological measures were defined. EEG recordings from one (male) participant were excluded due to poor quality (Fig. 3). *Region of interest analyses:* The regions of interest (ROI) i.e., sgACC-OFC, right insula, left insula, right amygdala, and left amygdala, were defined using the ‘ROI maker 3’ function in standardized low-resolution brain electromagnetic tomography (sLORETA). The ROI were specified by providing a seed point, and all voxels within a radius of 20 mm (sgACC-OFC, 0, 26, −10), 10 mm (insulae, 40, 18, −12 and −40, 18, −12), or 15 mm (amygdalae, 20, −2, −20 and −20, −2, −20) were averaged to calculate the log-transformed current density. *Functional connectivity analyses:* Lagged linear connectivity was used as a measure of coherence between the ROI in the aforementioned frequency bands and estimated using sLORETA.

### Statistical analysis

Statistical analyses were performed using R version4.3.0 (R Core Team, Vienna, Austria, 2021; www.R-project.org/), RStudio version2023.06.0 + 421 (RStudio team, Boston, MA, USA, 2020; http://www.rstudio.com/), and GraphPad Prism version10.2.1 (GraphPad Software, Boston, Massachusetts USA, www.graphpad.com). The significance threshold was *α* = 0.05 with Bonferroni-corrections applied to clinical (0.05/8, *α* = 0.006), and physiological outcome measures (0.05/50, *α* = 0.001).

#### Demographic variables and clinical outcome assessments

Descriptive statistics were used to identify group differences at T0 and participant evaluations of treatment. Thereafter, clinical outcomes of HD-tIGNS as a treatment for GAD were analyzed. Firstly, percentage change between time points (T0-T1, T1-T2) were calculated. Then, to examine within and between group differences, Wilcoxon Signed Rank and Mann Whitney U tests were used respectively. Participants with anxiety score changes greater than the Minimal Clinically Important Difference (MCID) from T0-T1 were also investigated (GAD-7: ≥ 4-point decrease;^72^ HADSA: ≥ 1.7-point decrease;^76^ STAI-T: ≥ 8-point decrease)^77^.

#### Physiological outcome assessments

Resting-state EEG collected at T0, T1, and T2 provided unbiased measurements used to explore the effects of HD-tIGNS on brain activity in, and FC between the sgACC-OFC, insulae, and amygdalae. All physiological measures were Bonferroni corrected for multiple comparisons (i.e., five brain regions: 0.05/5, *α* = 0.01). Mann Whitney U tests compared the Z score (Z = ((observed value-sample mean)/sample standard deviation)) change between ES and DS groups from T0-T1 (i.e., between-subjects treatment vs control) and Wilcoxon Signed Rank tests compared the Z score change between T0-T1 and T1-T2 in the DS group (i.e., within-subjects treatment vs control).

## Results

### Sample description

A total of *N* = 22 participants (*F* = 21(96%)) completed the study and were randomized equally into ES(*n* = 11) or DS(*n* = 11) groups. A total of *n* = 2 participants dropped out for reasons unrelated to the study (Fig. 3). For descriptive statistics at T0, see Table 1 and Supplemental Table S1.

**Table 1:** Sample demographics and clinical characteristics.

Group	Early start *N* = 11	Delayed start *N* = 11
Demographic		
Sex (male/female), *n* (%)	0/11 (0/100)	1/10 (9/91)
Age, mean ± SD	24 ± 5	28 ± 10
Ethnicity		
NZ European, *n* (%)	8 (73)	2 (18)
Māori, *n* (%)	1 (9)	3 (27)
English, *n* (%)	–	1 (9)
Chinese, *n* (%)	1 (9)	3 (27)
Filipino, *n* (%)	–	1 (9)
Taiwanese, *n* (%)	–	1 (9)
Indian, *n* (%)	1	–
Baseline questionnaire scores		
Generalized anxiety disorder- 7 (GAD-7), mean ± SD	14 ± 4	14 ± 4
Hospital anxiety and depression scale-anxiety (HADSA), mean ± SD	14 ± 2	13 ± 4

### Feasibility, treatment safety, and participant evaluation

#### Feasibility

The total recruitment period was four months (February–May 2023) with an average recruitment rate of six participants per month. The final assessment session and conclusion of the trial was October 2023 with the desired number of participants recruited. The proportion of participants recruited (24) from the total number screened (84) was 29%. The main reasons for participants not enrolling were not wanting to participate (20.7%), no response (16.1%), or having one or more exclusion criteria (14.9%). Of the participants recruited a dropout rate of 8.3% was reported. Overall, mean treatment adherence excluding dropouts was 99.5% (ES = 97%, DS = 98%). Blinding was successful whereby the treatment group was not revealed to participants. In total, 45% of participants reported they did not know which treatment group they were in and 18% guessed it incorrectly. Of the 36% of participants who guessed correctly, not one was 100% confident with their guess.

#### Safety and participant evaluation

**Fig. 4:**
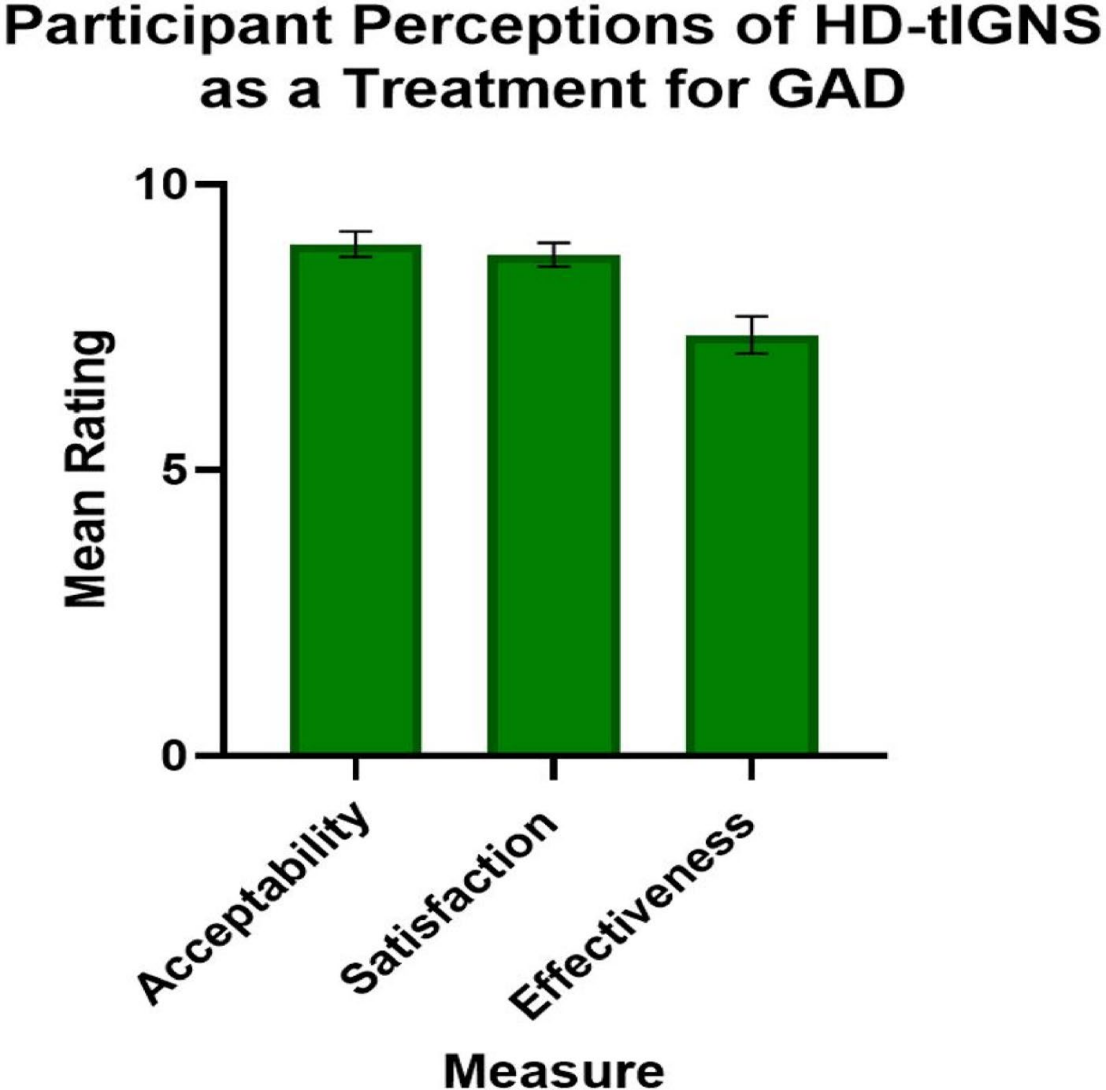
Participant evaluations of the acceptability, effectiveness, and satisfaction (on the x axis) of HD-tIGNS as a treatment for GAD. Mean participant ratings (on the y axis) range from 0–10 where 0 = not at all, and 10 = extremely. Standard error of measurement bars are shown for each aspect. Participants rated HD-tIGNS as a highly effective treatment.

The distribution of reported adverse effects of HD-tIGNS identified using the DESS Questionnaire (Table 2), showed the most common selected symptom was ‘increased dreaming or nightmares’ observed in the DS group at T1. No serious effects were observed. Overall, participants rated HD-tIGNS as a highly acceptable and effective treatment (Fig. 4).

**Table 2:** Participant responses for the discontinuation-emergent sign and symptom (DESS) questionnaire.

Symptom	T1 treatment (*n* = 11)	T1 Actisham (*n* = 11)	T2 (*N* = 22)
Elevated mood, feeling high	9%	9%	
Irritability	9%		
Confusion or trouble concentrating	9%		
Forgetfulness, memory problems	9%		4.5%
Trouble sleeping, insomnia		9%	4.5%
Increased dreaming or nightmares	9%	27%	9%
Fatigue, tiredness		9%	9%
Headache	9%		4.5%
Dizziness, light-headedness, or sensation of spinning (vertigo)	9%		
Chills	9%		4.5%
Stomach cramps		9%	
Burning, numbness, tingling sensations	9%		
Nose running			4.5%
Unusual visual sensations (lights, colors, geometric shapes)			4.5%

### Clinical measures *(exploratory)*

**Fig. 5:**
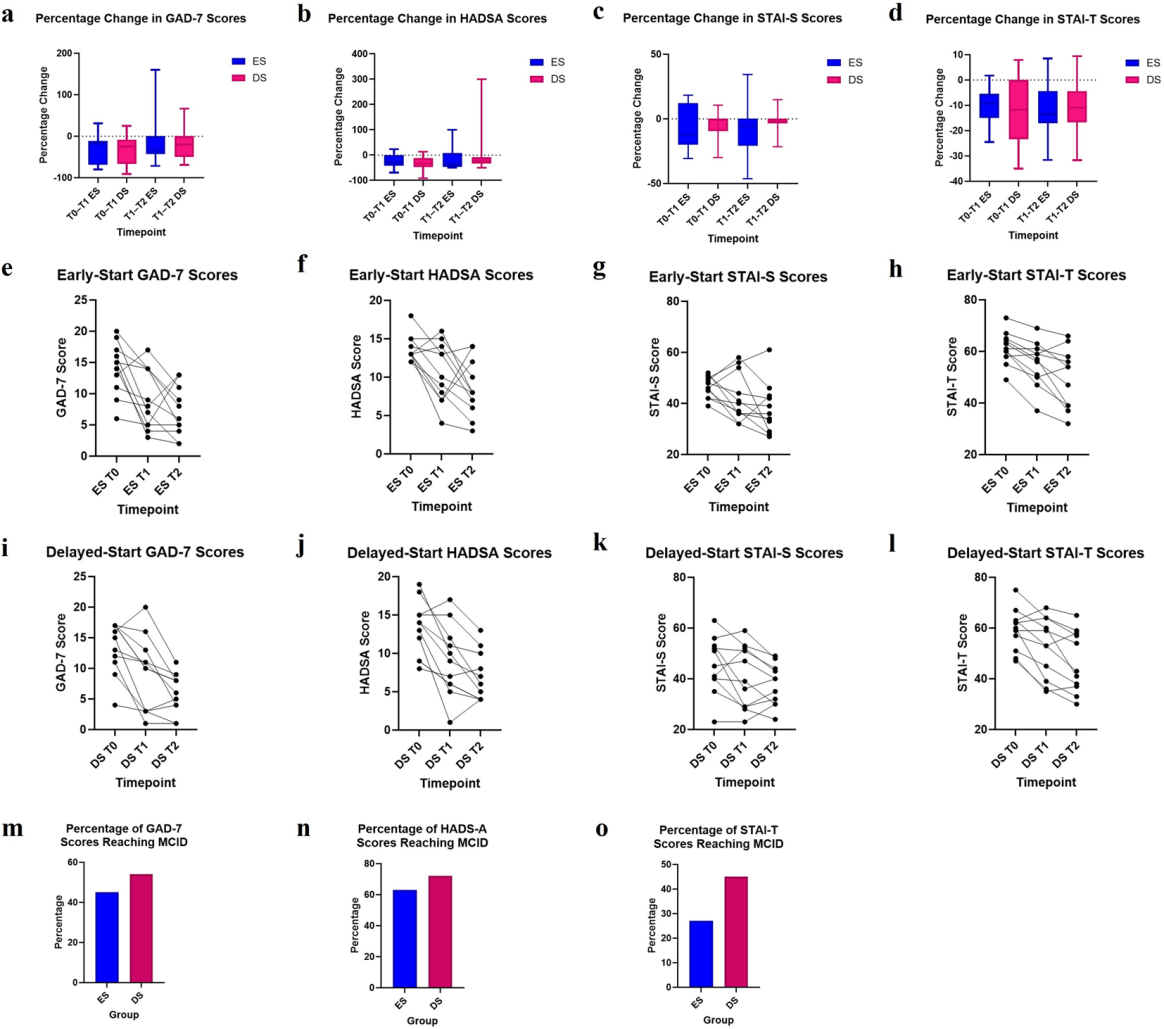
Shown is the percentage change in clinical outcome measures from T0-T1 and T1-T2 for the ES (blue) and DS (magenta) groups, specifically for the GAD-7 (**a**), HADSA (**b**), STAI-S (**c**), and STAI-T (**d**). Also shown are the individual participant total scores at timepoints T0, T1, and T2, specifically for the ES group: GAD-7 (**e**) HADS-A (**f**), STAI-S (**g**) and STAI-T (**h**), and the DS group: GAD-7 (**i**) HADS-A (**j**), STAI-S (**k**), and STAI-T (**l**). Moreover, the percentage of the ES and DS groups who met MCID thresholds at T1 are shown for the GAD-7 (**m**), HADSA (**n**), and STAI-T (**o**). These graphs explore possible clinical effects of HD-tIGNS on anxiety scores across groups and timepoints. While this study was not powered for hypothesis testing, no significant differences in clinical outcomes in the current sample were observed.

Mann Whitney U tests investigating group differences in percentage change from T0-T1 showed no significant differences between ES and DS groups for the GAD-7 (median = ES −25% DS -18%, *n*_1_ = *n*_2_ = 11, *U* = 58.5, *p* = 0.91), HADSA (median = ES -28% DS -33%, *n*_1_ = *n*_2_ = 11, *U* = 52, *p* = 0.59), STAI-S (median = ES -11.9% DS -2.5%, *n*_1_ = *n*_2_ = 11, *U* = 58, *p* = 0.898), and STAI-T (median = ES -9.2% DS -11.8%, *n*_1_ = *n*_2_ = 11, *U* = 52.5, *p* = 0.619). Wilcoxon Signed Rank tests investigating within-group differences in percentage change in the DS group (T0-T1 vs T1-T2), showed no significant differences for GAD-7 (*W*(11) = 7, *p* = 0.75), HADSA (*W*(11) = 11, *p* = 0.57), STAI-S (*W*(11) = 12, *p* = 0.638), or STAI-T (*W*(11) = −8, *p* = 0.741). Clinical outcome scores that met MCID thresholds demonstrated a considerable percentage of participants from both groups for GAD-7: ES = 45% (*n* = 5), DS = 54% (*n* = 6); HADSA:ES = 63% (*n* = 7), DS = 72% (*n* = 8); STAI-T: ES = 27% (*n* = 3), DS = 45% (*n* = 5)(Fig. 5). Furthermore, in the total sample from T0-T2 82%(*n* = 18) of participants had GAD-7 scores that normalized (i.e., score ≤ 9).

### Physiological measures *(exploratory)*

**Fig. 6:**
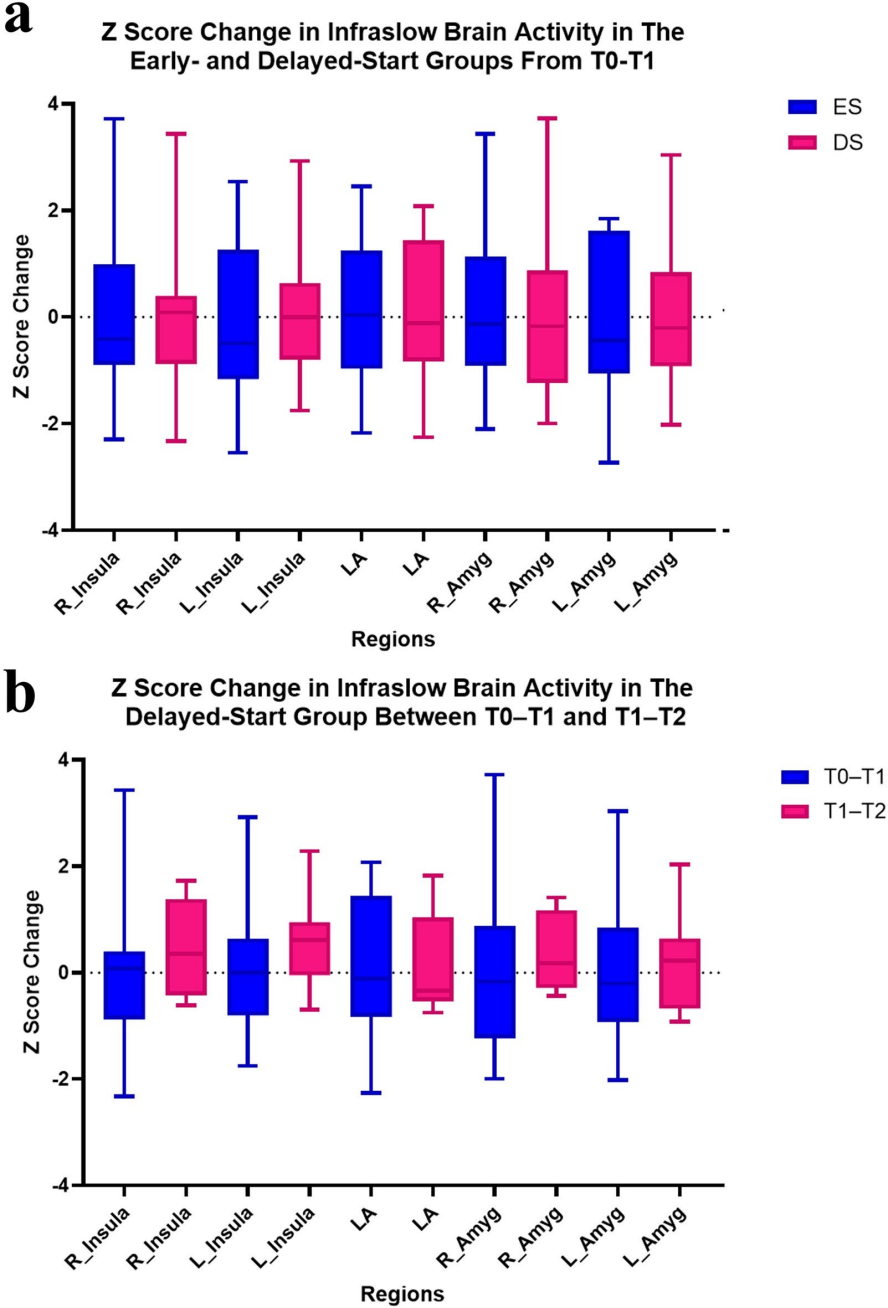
Shown are the Z score changes for the physiological outcome measure infraslow brain activity*. While this study was not powered for hypothesis testing, these graphs show that high definition transcranial infraslow gray noise stimulation (HD-tIGNS) displayed no significant differences in brain activity between the ES and DS groups from T0-T1 (**a**), or in the DS group when comparing T0-T1 vs T1-T2 (**b**). LA = Large Area (= sgACC-OFC). * EEG recordings from one participant in the DS group were excluded from all analyses due to poor quality.

**Fig. 7:**
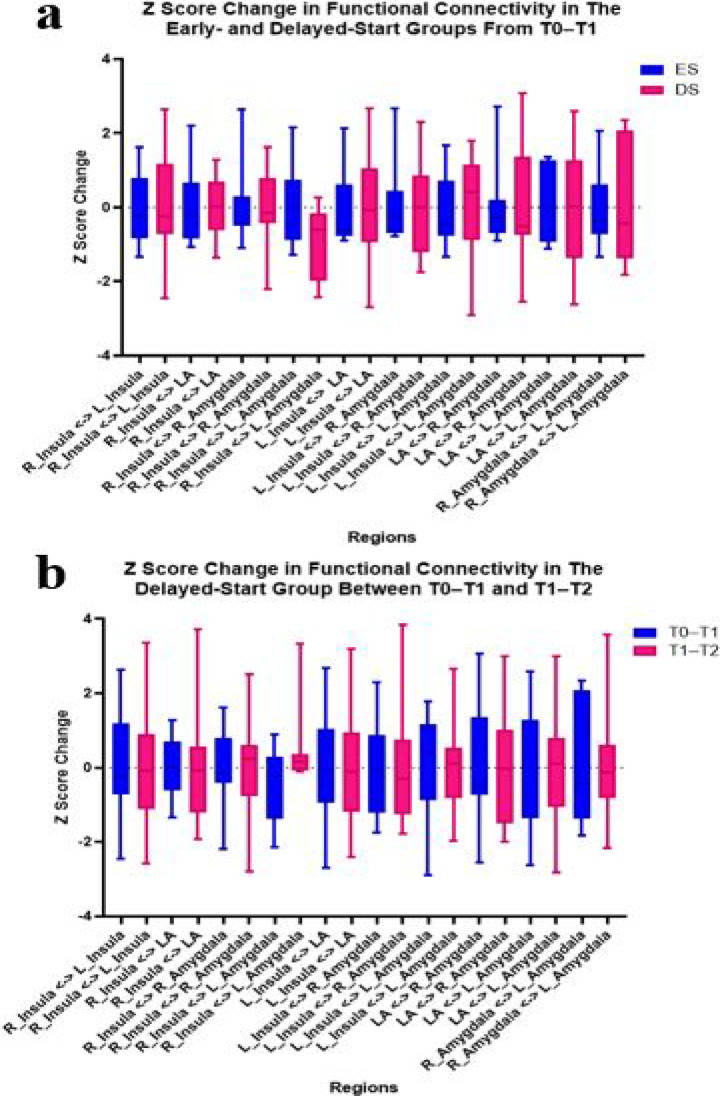
Shown are the Z score changes for the physiological outcome measure infraslow functional connectivity*. While this study was not powered for hypothesis testing, these graphs show that high definition transcranial infraslow gray noise stimulation (HD-tIGNS) displayed no significant effect on functional connectivity between the sgACC-OFC, insulae, or amygdalae, between the ES and DS groups from T0-T1 (**a**) and within the DS group comparing T0-T1 vs T1-T2 (**b**). LA = Large Area (= sgACC-OFC). * EEG recordings from one participant in the DS group were excluded from all analyses due to poor quality.

Brain activity (i.e., current source density) in the infraslow frequency band was investigated using Mann Whitney U tests to compare the Z score changes between ES and DS groups from T0-T1. No significant differences in the sgACC-OFC, insulae, or amygdalae were observed (Fig. 6a). Furthermore, Wilcoxon Signed Rank tests showed no significant differences in infraslow brain activity in the DS group between T0-T1 and T1-T2 (Fig. 6b). For infraslow FC between the sgACC-OFC, insulae, and amygdalae, Mann Whitney U tests show no significant differences between ES and DS groups from T0-T1 (Fig. 7a), and Wilcoxon Signed Rank tests showed no significant differences in the DS group comparing T0-T1 vs T1-T2 (Fig. 7b).

To test whether the infraslow or gray noise component was responsible for the observed clinical and physiological outcomes, analyses on five further frequency bands (delta (2–3.5 Hz), theta (4–7.5 Hz), alpha (8–12 Hz), beta (12.5–30 Hz), and gamma (30.5–44 Hz)) were investigated in the sgACC-OFC, insulae, and amygdalae. Mann Whitney U tests comparing the percentage change between ES and DS groups from T0-T1, and Wilcoxon Signed Rank tests investigating differences in percentage change in the DS group between T0-T1 and T1-T2 showed no significant differences in activity or FC in any frequency bands (Supplemental Tables S2 and S3).

## Discussion

The aim of this study was to evaluate the feasibility and safety of HD-tIGNS in individuals with GAD and explore potential effects of HD-tIGNS on clinical anxiety outcomes, and brain activity and FC. The low drop-out rates, high treatment adherence, and absence of serious adverse effects observed in this study suggest that as expected, HD-tIGNS is a feasible, and safe treatment for GAD. Interestingly, the most common reported adverse effect was ‘increased dreaming or nightmares’. This effect has been observed in other studies investigating neuromodulatory techniques such as neurofeedback, and has been suggested to be an indication of brain learning, or brain response to treatment^78,79^. Dreaming requires engagement of multiple brain regions and it is thought that these regions were challenged during awake states, meaning that dreaming/nightmares can represent a neuroplastic adaptation to events occurring while awake^79^. Interestingly, at T1 this adverse effect was most commonly reported in the DS group, whereas it would be expected to be more frequently observed in the ES group. The reasons for this unexpected finding might include: (1) Actisham stimulation reaches and affects different brain regions to HD-tIGNS. (2) A non-linear response to treatment is occurring such that the very short stimulation period applied during actisham stimulation provokes increased dreaming/nightmares, whereas the HD-tIGNS stimulation applied may see the brain habituate or adapt during the stimulation period itself rather than during sleep. 93) Considering the small sample size this may be a statistical anomaly. In sum, this observation serves as a reminder of the complex interplay between the brain and neuromodulatory intervention and suggests the actisham stimulation might introduce effects which complicate interpretation of the findings.

It is important to note this study was not powered to test for efficacy and secondary outcomes (i.e., clinical and physiological outcomes) should be interpreted with caution. However, it is intriguing that no significant differences in clinical outcomes between the treatment and control groups were observed. Because it is well known that treatments for GAD are characterized by moderate to large placebo effects, analogous to what is found in mental disorders in general^80,81^, this may be due to a placebo effect. However, the possibility needs to be excluded that actisham stimulation might have induced a minimal-dose effect whereby some of the shunted current might have reached the brain^70^. While no significant differences within or between groups were observed, around 60% of both the ES and DS groups met the threshold for MCID in the GAD-7 and HADS-A, and 45% of the DS group for the STAI-T. Thus, we hypothesize that MCIDs observed in the DS group from T0-T1 were not simply placebo effects^82^, but rather nonspecific effects of actisham stimulation^70^. Despite actisham only applying small E-fields to the brain targets, non-linear amplification effects of actisham are possible. Evidence supporting this hypothesis can be found in existing literature which finds similar minimal-dose effects using burst stimulation^83–86^. Because actisham uses the same infraslow gray noise stimulation waveform but with much lower E-fields, actisham might still reach the targeted brain areas resulting in a minimal-dose effect that influenced the clinical outcomes observed in this study. Moreover, the proportion of participants in the DS group who reached MCID matches existing literature which observes that ~ two-thirds of participants respond to stimulatory treatments, leaving one-third of non-responders^43^. Therefore, it is essential to further investigate actisham stimulation compared to a placebo group receiving no current. If minimal-dose stimulation can harbor similar results to standard stimulation, then time and equipment would allow for more patients to be treated.

The hypothesized effect of HD-tIGNS to alter pathological activity and FC between the key hubs of the anxiety network (sgACC-OFC, insulae, amygdalae) showed no significant differences in functional connectivity between the right insula and sgACC-OFC. The absence of changes in both brain activity and FC indicates that HD-tIGNS might not affect the anxiety network as predicted, such that pathological EEG signals were not altered toward more neurotypical activity patterns. The infraslow frequency has previously been observed to be disrupted in anxiety disorder patients and has been found to coordinate and integrate information within connectivity networks by modulating excitability^18,20,87^. Hence, the HD-tIGNS protocol used in this study might only reach superficial brain areas and not the deeper brain regions that HD-tIGNS was intended to reach. Because a large proportion of participants in both groups achieved MCID and had GAD-7 scores that normalized, it is possible that HD-tIGNS only reached superficial brain areas. This may have been sufficient to induce clinical improvements in anxiety patients. It is recommended that future research investigates stimulation protocols of different amplitude intensities and targets different ROI.

The use of high-definition stimulation was a major strength of this study as it allowed for precise stimulation of the targeted regions compared to other methods^58,88^. Limitations of this study include firstly, only one participant was male which might add to group differences and limit generalizability to males affected with GAD. The physiological outcome measure for the male participant was lost due to poor recording quality. Therefore, any interpretations of the physiological outcome measures must be solely with respect to females (See Fig. 3). Secondly, practical issues might have influenced the effect of treatment, i.e., eight participants had health related (e.g., COVID-19) postponements of multiple sessions during the six-week period, and many participants had stimulations on irregular times and days throughout the trial. These limitations should be carefully controlled in future research.

This study provides preliminary observations exploring the effects of HD-tIGNS as a novel technique which, based on the results of this study, is a safe and feasible potential treatment approach for GAD. While no significant changes in brain activity or FC were observed in the sgACC-OFC, insulae, or amygdalae, participants across groups and timepoints reported substantial improvements in clinical outcomes, although these findings are likely attributable to strong placebo responses as no significant differences between groups were observed. Future investigation into HD-tIGNS might revise the targeted brain regions as a potential treatment for GAD.

## Supplementary Information

Below is the link to the electronic supplementary material.

Supplementary Material 1

Supplementary Material 2

## Data Availability

The data that support the findings of this study are available from the corresponding author upon request.
