# Supplementary material for: Investigating the feasibility and safety of transcranial infraslow gray noise stimulation as a potential treatment for generalized anxiety disorder
Source: Sci Rep. 2025 Dec 17;15:43975. doi: 10.1038/s41598-025-27624-3 (PMC12712071; doi:10.1038/s41598-025-27624-3)
Supplement: Supplementary file 1 — Supplementary Material 1 [file 41598_2025_27624_MOESM1_ESM.docx]

**SUPPLEMENTAL MATERIAL**

**Fig S.1** Inclusion and exclusion criteria

**Table S.1**

*Sample demographics continued*

| Table S.1. Sample Demographics | | |
| --- | --- | --- |
| **Group** | **Early Start**  ***N*= 11** | **Delayed Start**  ***N*= 11** |
| **Employment** |  |  |
| Student, *n* (%) | 5 (46) | 5 (46) |
| Employed, *n* (%)  Employed, student | 5 (46)  - | 3 (27)  2 (18) |
| Unemployed, *n* (%)  Unemployed, looking after family | 1 (9)  - | -  1 (9) |
| **Education** |  |  |
| Certificate/diploma, *n* (%)  Year 12/equivalent, *n* (%)  Trade/Apprenticeship, *n* (%) | -  4 (36)  1 (9) | 1(9)  7 (64)  - |
| University degree, *n* (%)  No formal education, *n* (%) | 6 (55)  - | 2 (18)  1 (9) |
| **Medication** |  |  |
| Sertraline, *n* (%) | 2 (18) | 3 (27) |
| Escitalopram, *n* (%) | 3 (27) | 2 (18) |
| Fluoxetine, *n* (%)  Venlafaxine, *n* (%) | 4 (36)  - | 1 (9)  3 (27) |
| Oral contraceptives, *n* (%) | 3 (27) | 1 (9) |
| No medication, *n* (%) | 2 (18) | 2 (18) |
|  |  |  |

**Table S.2.**

*Post Hoc Comparison of Infraslow and Noise Components of HD-tIGNS; Activity*

| Table S.2. Post Hoc Comparison of Infraslow and Noise Components of HD-tIGNS; Activity | | | | | |
| --- | --- | --- | --- | --- | --- |
| **Physiological Outcome** | **Frequency Band Statistics** (Mann Whitney U tests for Between Group, and Two tailed Wilcoxon Signed Rank Tests for Within Group Analyses) | | | | |
| **Activity ES vs DS**  Right Insula  Left Insula  sgACC-OFC  Right Amygdala  Left Amygdala  **Activity DS T0–T1** **vs DS T1–T2**  Right Insula  Left Insula  sgACC-OFC  Right Amygdala  Left Amygdala | **Delta**  (*U*= 51, *p*= 0.809)  (*U*= 53, *p*= 0.918)  (*U*= 54, *p*= 0.973)  (*U*= 50, *p*= 0.756)  (*U*= 53, *p*= 0.918)  (*W*(10)= 1, *p*= >0.999)  (*W*(10)= -1, *p*= >0.999)  (*W*(10)= 9, *p*= 0.695)  (*W*(10)= 1, *p*= >0.999)  (*W*(10)= 5, *p*= 0.846) | **Theta**  (*U*= 55, *p*= >0.999)  (*U*= 52, *p*= 0.863)  (*U*= 54, *p*= 0.973)  (*U*= 53, *p*= 0.918)  (*U*= 53, *p*= 0.918)  (*W*(10)= -3, *p*= 0.922)  (*W*(10)= 3, *p*= 0.922)  (*W*(10)= 3, *p*= 0.922)  (*W*(10)= -1, *p*= >0.999)  (*W*(10)= -1, *p*= >0.999) | **Alpha**  (*U*= 54, *p*= 0.973)  (*U*= 53, *p*= 0.918)  (*U*= 50, *p*= 0.756)  (*U*= 50, *p*= 0.756)  (*U*= 53, *p*= 0.918)  (*W*(10)= -1, *p*= >0.999)  (*W*(10)= 5, *p*= 0.846)  (*W*(10)= 1, *p*= >0.999)  (*W*(10)= 1, *p*= >0.999)  (*W*(10)= -1, *p*= >0.999) | **Beta**  (*U*= 51, *p*= 0.809)  (*U*= 53, *p*= 0.918)  (*U*= 48, *p*= 0.654)  (*U*= 53, *p*= 0.918)  (*U*= 55, *p*= >0.999)  (*W*(10)= 1, *p*= >0.999)  (*W*(10)= 9, *p*= 0.695)  (*W*(10)= 3, *p*= 0.922)  (*W*(10)= -3, *p*= 0.922)  (*W*(10)= 9, *p*= 0.695) | **Gamma**  (*U*= 54, *p*= 0.973)  (*U*= 52, *p*= 0.863)  (*U*= 49, *p*= 0.705)  (*U*= 53, *p*= 0.918)  (*U*= 54, *p*= 0.973)  (*W*(10)= 1, *p*= >0.999)  (*W*(10)= 1, *p*= >0.999)  (*W*(10)= 1, *p*= >0.999)  (*W*(10)= 1, *p*= >0.999)  (*W*(10)= 7, *p*= 0.770) |

**Table S.3.**

*Post Hoc Comparison of Infraslow and Noise Components of HD-tIGNS; Functional Connectivity*

| Table S.3. Post Hoc Comparison of Infraslow and Noise Components of HD-tIGNS; Functional Connectivity | | | | | |
| --- | --- | --- | --- | --- | --- |
| **Physiological Outcome** | **Frequency Band Statistics** (Mann Whitney U tests for Between Group, and Two tailed Wilcoxon Signed Rank Tests for Within Group Analyses) | | | | |
| **Functional Connectivity ES vs DS**  Right Insula <-> Left Insula  Right Insula <-> sgACC-OFC  Right Insula <-> Right Amygdala  Right Insula <-> Left Amygdala  Left Insula <-> sgACC-OFC  Left Insula <-> Right Amygdala  Left Insula <-> Left Amygdala  sgACC-OFC <-> Right Amygdala  sgACC-OFC <-> Left Amygdala    Right Amygdala <-> Left Amygdala  **Functional Connectivity DS T0–T1** **vs DS T1–T2**  Right Insula <-> Left Insula  Right Insula <-> sgACC-OFC  Right Insula <-> Right Amygdala  Right Insula <-> Left Amygdala  Left Insula <-> sgACC-OFC  Left Insula <-> Right Amygdala  Left Insula <-> Left Amygdala  sgACC-OFC <-> Right Amygdala  sgACC-OFC <-> Left Amygdala    Right Amygdala <-> Left Amygdala | **Delta**  (*U*= 54, *p*= 0.973)  (*U*= 51, *p*= 0.810)  (*U*= 51, *p*= 0.810)  (*U*= 53, *p*= 0.918)  (*U*= 51, *p*= 0.810)  (*U*= 47, *p*= 0.605)  (*U*= 55, *p*= >0.999)  (*U*= 49, *p*= 0.720)  (*U*= 50, *p*= 0.756)  (*U*= 51, *p*= 0.810)  (*W*(10)= -16, *p*= 0.520)  (*W*(10)= 10, *p*= 0.700)  (*W*(10)= -14, *p*= 0.577)  (*W*(10)= -28, *p*= 0.240)  (*W*(10)= 12, *p*= 0.638)  (*W*(10)= -30, *p*= 0.206)  (*W*(10)= -10, *p*= 0.700)  (*W*(10)= -26, *p*= 0.278)  (*W*(10)= 0, *p*= >0.999)  (*W*(10)= -30, *p*= 0.206) | **Theta**  (*U*= 47, *p*= 0.605)  (*U*= 55, *p*= >0.999)  (*U*= 50, *p*= 0.756)  (*U*= 54, *p*= 0.973)  (*U*= 52, *p*= 0.863)  (*U*= 54, *p*= 0.973)  (*U*= 54, *p*= 0.973)  (*U*= 54, *p*= 0.973)  (*U*= 51, *p*= 0.810)  (*U*= 50, *p*= 0.756)  (*W*(10)= -20, *p*= 0.413)  (*W*(10)= -6, *p*= 0.831)  (*W*(10)= -18, *p*= 0.465)  (*W*(10)= -18, *p*= 0.465)  (*W*(10)= -36, *p*= 0.123)  (*W*(10)= -38, *p*= 0.102)  (*W*(10)= -48, *p*= 0.032)  (*W*(10)= -8, *p*= 0.765)  (*W*(10)= -14, *p*= 0.577)  (*W*(10)= -26, *p*= 0.278) | **Alpha**  (*U*= 52, *p*= 0.863)  (*U*= 53, *p*= 0.918)  (*U*= 54, *p*= 0.973)  (*U*= 48, *p*= 0.654)  (*U*= 51, *p*= 0.810)  (*U*= 52, *p*= 0.863)  (*U*= 49, *p*= 0.720)  (*U*= 50, *p*= 0.756)  (*U*= 55, *p*= >0.999)  (*U*= 55, *p*= >0.999)  (*W*(10)= -22, *p*= 0.365)  (*W*(10)= 12, *p*= 0.638)  (*W*(10)= 44, *p*= 0.054)  (*W*(10)= -16, *p*= 0.520)  (*W*(10)= -8, *p*= 0.765)  (*W*(10)= -30, *p*= 0.206)  (*W*(10)= -12, *p*= 0.638)  (*W*(10)= 6, *p*= 0.831)  (*W*(10)= -10, *p*= 0.700)  (*W*(10)= -16, *p*= 0.520) | **Beta**  (*U*= 52, *p*= 0.863)  (*U*= 49, *p*= 0.720)  (*U*= 50, *p*= 0.756)  (*U*= 53, *p*= 0.918)  (*U*= 54, *p*= 0.973)  (*U*= 44, *p*= 0.468)  (*U*= 50, *p*= 0.756)  (*U*= 53, *p*= 0.918)  (*U*= 47, *p*= 0.605)  (*U*= 48, *p*= 0.654)  (*W*(10)= 18, *p*= 0.465)  (*W*(10)= -22, *p*= 0.365)  (*W*(10)= 16, *p*= 0.520)  (*W*(10)= 12, *p*= 0.638)  (*W*(10)= -2, *p*= 0.966)  (*W*(10)= 16, *p*= 0.520)  (*W*(10)= 22, *p*= 0.365)  (*W*(10)= 10, *p*= 0.700)  (*W*(10)= 6, *p*= 0.831)  (*W*(10)= 10, *p*= 0.700) | **Gamma**  (*U*= 48, *p*= 0.654)  (*U*= 48, *p*= 0.654)  (*U*= 52, *p*= 0.863)  (*U*= 50, *p*= 0.756)  (*U*= 51, *p*= 0.810)  (*U*= 53, *p*= 0.918)  (*U*= 54, *p*= 0.973)  (*U*= 54, *p*= 0.973)  (*U*= 53, *p*= 0.918)  (*U*= 53, *p*= 0.918)  (*W*(10)= -38, *p*= 0.102)  (*W*(10)= -12, *p*= 0.638)  (*W*(10)= 34, *p*= 0.148)  (*W*(10)= -30, *p*= 0.206)  (*W*(10)= -8, *p*= 0.765)  (*W*(10)= -18, *p*= 0.465)  (*W*(10)= 12, *p*= 0.638)  (*W*(10)= 38, *p*= 0.102)  (*W*(10)= -2, *p*= 0.966)  (*W*(10)= -32, *p*= 0.175) |
