# Supplementary figures and images for: Investigating the feasibility and safety of transcranial infraslow gray noise stimulation as a potential treatment for generalized anxiety disorder
Source: Sci Rep. 2025 Dec 17;15:43975. doi: 10.1038/s41598-025-27624-3 (PMC12712071; doi:10.1038/s41598-025-27624-3)

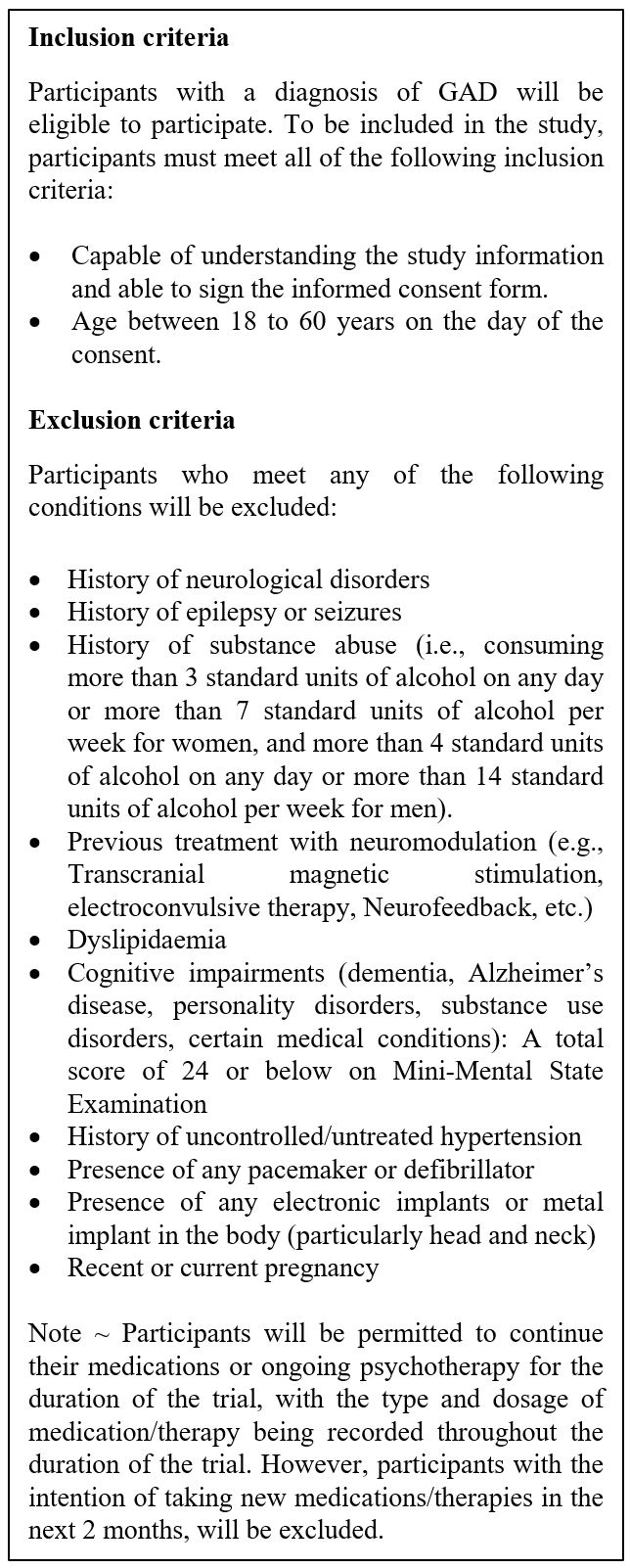

Supplement: Supplementary file 2 — Supplementary Material 2 [file 41598_2025_27624_MOESM2_ESM.jpg]
